# PypeTree: A Tool for Reconstructing Tree Perennial Tissues from Point Clouds

**DOI:** 10.3390/s140304271

**Published:** 2014-03-04

**Authors:** Sylvain Delagrange, Christian Jauvin, Pascal Rochon

**Affiliations:** Institute of Temperate Forest Sciences (ISFORT), University of Quebec in Outaouais (UQO), 58 Rue Principale, Ripon, QC J0V1V0, Canada; E-Mails: cjauvin@gmail.com (C.J.); rochon.pascal@uqo.ca (P.R.)

**Keywords:** Terrestrial LiDAR Scanning (TLS), tree reconstruction, skeleton, L-System, validation procedure, colonisation algorithm, botanical trees

## Abstract

The reconstruction of trees from point clouds that were acquired with terrestrial LiDAR scanning (TLS) may become a significant breakthrough in the study and modelling of tree development. Here, we develop an efficient method and a tool based on extensive modifications to the skeletal extraction method that was first introduced by Verroust and Lazarus in 2000. PypeTree, a user-friendly and open-source visual modelling environment, incorporates a number of improvements into the original skeletal extraction technique, making it better adapted to tackle the challenge of tree perennial tissue reconstruction. Within PypeTree, we also introduce the idea of using semi-supervised adjustment tools to address methodological challenges that are associated with imperfect point cloud datasets and which further improve reconstruction accuracy. The performance of these automatic and semi-supervised approaches was tested with the help of synthetic models and subsequently validated on real trees. Accuracy of automatic reconstruction greatly varied in terms of axis detection because small (length < 3.5 cm) branches were difficult to detect. However, as small branches account for little in terms of total skeleton length, mean reconstruction error for cumulated skeleton length only reached 5.1% and 1.8% with automatic or semi-supervised reconstruction, respectively. In some cases, using the supervised tools, a perfect reconstruction of the perennial tissue could be achieved.

## 1. Introduction

Terrestrial LiDAR Scanning (TLS) measures the precise location of objects in 3D space and, consequently, represents a promising tool for studying large and complex organisms such as trees [1,2]. In recent years, it has become an efficient alternative to established forest inventory methods for obtaining data on forest structure [3–5] and has also been used to extract whole-tree or crown traits from a tree or a group of trees [6–9]. Because trees are generally tall and have complex crowns, *in situ* measurements and analysis of structural attributes are often difficult, if not impossible, to perform. While a limited number of fixed and mobile canopy cranes exist, ground-based methods are still needed, as they are more practical. When coupled with plant modelling, realistic reconstructions of botanical trees can substantially improve the quality of functional plant models [10]. Furthermore, incorporating realistic 3D structures into modelling efforts could lead to significant advances in our understanding of plant functional ecology and performance [11–13]. Moreover, studies using virtual tree models that have been developed from TLS data result in improved realistic surveys. Such models indeed include the imprint that is left by all real past stochastic events on the studied tree. However, significant work is still needed to obtain automated and reliable methods able to exploit such data, especially in an ecological context [14].

Several recent studies have devised algorithms to perform tree reconstruction from TLS-acquired point clouds [8,9,15–19]. Although these studies have yielded promising results (from saplings to adult trees), empirical validation of these tools is rarely performed. The issue of validating the reconstructed models is quite difficult in itself, given the size, the 3D arrangement, and the complexity of real trees. As a consequence, only a few studies have investigated quantitative methods for doing so [8,9,17]. Moreover, several challenges still need to be addressed to improve the completeness of models that result from imperfect point cloud data [19], especially for botanical and developmental studies. For instance, foliage reconstruction still needs to be performed using L-system-based modelling [16,17,20] or allometric relationships [8]. L-Systems may be more appropriate for larger trees for which the exact position of photosynthetic tissues is not the most important criterion, as compared to foliage density or total surface [21]. However, the precise reconstruction of the main skeleton, including small- and medium-size branches, remains crucial for displaying an appropriate distribution of foliage [17]. Conversely, allometric relationships are well suited for displaying foliage surfaces within individual crown, but this is only possible when skeleton reconstruction meets very high standards. To provide an efficient and multi purposes tool, we have therefore created PypeTree, an open-source modelling environment that is focused upon the full reconstruction of tree perennial tissues, which are the basis for adequate botanical tree reconstruction. The PypeTree reconstruction engine is based on the skeletal extraction algorithm that was introduced by Verroust and Lazarus [22], which we have extensively studied to devise a series of improvements. We have specifically focused on making the algorithm better-suited to the task of botanical tree reconstruction. PypeTree also includes a tool to create L-system-based synthetic models, the surfaces of which can be sampled to produce relatively realistic datasets for validation and development purposes. Here, we describe the model interface and also validate the accuracy of PypeTree reconstructions against actual field measurements taken from several sapling tree species.

## 2. Experimental Section

**Figure A1.**
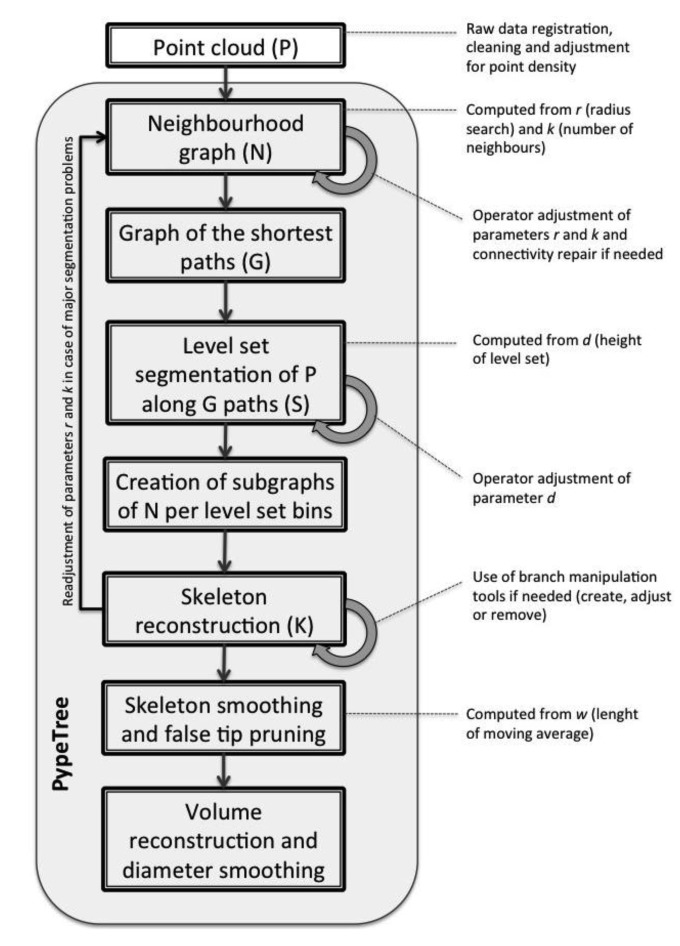
Conceptual diagram of the PypeTree approach.

The method of reconstruction in PypeTree is based on an algorithm that was devised by Verroust and Lazarus [22], and which allows the user to extract “skeletal curves” from 3D point clouds. This algorithm finds the implicit skeletal curve of a surface-sampled object by computing four distinct graph structures (N, G, S and K), each one of which is sequentially derived from the preceding structure. In this section, we will describe and discuss each component to highlight particular aspects that are relevant to the task of tree perennial tissue modelling in PypeTree (also see the conceptual diagram of PypeTree in Appendix material Figure A1).

### 2.1. The Neighbourhood Graph (N)

From the raw point cloud data (*P*), the original method computes the neighbourhood graph (*N*) with Euclidean proximity constraints in terms of a maximal number of neighbours (*k*). In original method of Verroust and Lazarus [22], the criterion for a suitable value of *k* is simply that the resulting graph should be fully connected. This may work well for simpler and more regularly shaped objects. However, for complex shapes like trees, we have found that it is better to compute *N* in terms of a search radius (*r*) for each point, without any limit on the number of neighbours (*k* = ∞). Given that both the sampling density (*i.e.*, the average distance between the points) and the minimal distance between the object features are known, this yields greater control over the form and integrity of the tree features that are encoded in the resulting connectivity graph. In the context of botanical tree reconstruction, we found that reliance upon a fixed number of neighbours easily leads to spurious connections between the trunk and small branches, which in turn causes problems later in the modeling process with respect to the structures that are derived from it. In contrast, using a search radius ensures that points separated by “gaps” shorter than the chosen distance will not be linked. This is of a special importance in our context, as botanical trees typically possess a dense network of small branches, which can be quite close to one another, especially as we reach higher into their canopy. In contrast to the original method, an optimal value for *r* in our setting does not necessarily imply a fully connected graph. As TLS datasets are prone to occlusion problems, some parts of the scene may be missing, which can result in a poorly connected graph if *r* is below a certain threshold. Although it might be tempting to augment the value of the connectivity parameters (*r*) to avoid this problem, it is actually preferable to begin with a model with some disconnected components to prevent subsequent false links between distinct small branches. In other words, maintaining a high value for *k* (*k* = ∞ being too time-consuming for calculations) to avoid spurious connection, together with the inclusion of connecting points that are based on a search radius (*r*) just below the minimum branch length to be detected, will produce the best results for *N*. The ideal connectivity structure that results from this first processing stage is one that optimizes the trade-off between graph density and the structural integrity of the features. This trade-off is very much dependent upon raw scan quality and, more specifically, upon occlusion. In cases where occlusion remains a great concern, some visual and interactive approaches might be considered (see “interactive adjustment tools” section) rather than increasing *r*.

### 2.2. The Geodesic Graph (G)

We compute the shortest path from every point in *N* to the source point using Dijkstra's shortest path algorithm [23]. In PypeTree, the source point is defined as the one with the lowest height coordinate, and corresponds to the base of the tree trunk. The resulting graph *G* (a sub-graph of *N*), along with the path edge distances, can be thought as a representation of the geodesic space of the tree surface, with the many dimensions spanning from the trunk source to the tips of the branches, along each path.

### 2.3. The Level Sets (S)

The original method defines the level sets *S* as a partitioning of *P* in *k*-quantized distance bins (in other words, the sets of points that are located at approximately constant distances from the source), which can be computed using the information that is contained in *G*. Because of the particular structure of trees, it makes more sense in our context to define the level sets (*d*) in terms of the minimal branch length. If *d* is greater than this real minimal length, it will not be possible to distinguish finer features, and segmentation resolution will not be sufficient. Conversely, if *d* is lower than the real minimal branch length, then segmentation resolution will possibly create false branches. Typically, near the base of a tree, the level sets will be associated with single cylinder cuts (*i.e.*, those composing the trunk). Higher up the trunk, they will tend to fragment into many branch cuts, which we will then need to segment. The segmentation of the particular level set *S*_i_ (with *S*_i_ ⊂ *P*) is accomplished by extracting the connected components of *N*_i_, *i.e.*, the sub-graph that was obtained by the intersection of *N* with the points in *S*_i_. Note these two differences between the original method and ours at this stage:

- We reuse information contained in *N* (*i.e.*, the point links based on distance) to facilitate the segmentation of sets of points at the same level. This step avoids the computation of additional level-specific neighbourhood graphs.
- Our level sets are extended within the geodesic space (whereas they are thin “rings” in the original method); this helps to stabilise the segmentation process, as a thin-level set is associated with a greater chance of misconnection because of its low density.

### 2.4. The Skeleton (K)

Once the branch cuts have been segmented into connected components, their centroids will constitute the nodes of the tree skeleton graph *K* (for clarity, note that *K* *⊄ P*, rather than by extension of any data structure that has been computed so far: *N*, *G* and *S*). The edges of this graph are formed by connecting the *K-*nodes at level *i* (corresponding to the centroids of segments found in *N*_i_) to the ones at the level below *i* – 1 by following the geodesic paths passing through them (*G* ∩ *S*_i_ ∩ *S*_i-1_). This downward direction constrains skeleton graph construction in a manner useful for botanical tree reconstruction. Indeed, a node at a certain level cannot be linked to more than one node a level below, which is not true in the upward direction.

### 2.5. Additional Modelling Features and Tools

To maximise its usefulness and efficiency in the context of tree perennial tissue reconstruction, we have also introduced the following set of modifications to the original method.

#### 2.5.1. False Tip Pruning

There is a maximal value for *d*, beyond which it is not possible to segment small features because of insufficient segmentation resolution (see also Section 2.3). However, a small value for *d* introduces some instability into the segmentation process. Reducing the size of the level set sub-graphs increases their chances of being misconnected, which then results in the creation of spurious *K-*nodes (*i.e.*, false branches). Given that *N* connectivity was optimised (by optimising the *r* parameter that was based on original point cloud quality and expected minimal branch length; see Section 2.1), it was not possible to augment *N*'s density to reduce the *d* parameter. In other words, there is a trade-off between the connectivity level, which must be low enough to prevent fusion of distinct features, and segmentation resolution, which must be high enough to distinguish between them. For cases where this optimal compromise is not obtainable, it is possible to mitigate these segmentation problems once the skeleton structure has been computed. Because it is a tree (*i.e.*, in the mathematical sense), the tip nodes of the skeleton are easily identifiable as those without child nodes. To these, we apply a test to detect the existence of *G* paths that lead to higher geodesic levels. If any such path is found, the node, together with all of its ancestors down to the next branching intersection, can be removed, as it is considered a “false tip”.

#### 2.5.2. Skeleton Smoothing

The combined effects of increasing branching resolution and using the pruning tool usually produce skeletons with broken lines, especially when scan quality of the original image is low (*i.e.*, when scans have very low point density). To counter this, we smooth the skeleton by using a moving average (of length *w*, a user-defined parameter), which is applied from the tip of every branch to the base of the trunk.

#### 2.5.3. Volume Reconstruction

The final step in perennial tissue reconstruction is the assignment of a radius value to every skeleton segment, to model its thickness. We do this by collecting the level set “starting points,” *i.e.*, the *G* path nodes that first cross the level boundaries, when going upward from the source. These subsets of points are arranged in approximate circles the plane of which is perpendicular to the local branch direction. The mean radius can thus be computed and used as the branch base radius. To represent the truncated cone form of tree segments, the radii of all segments are smoothed to generate the complete final model. Validation of branch diameters that were provided by PypeTree was performed against direct measurements that were obtained from a second study dealing with ice accretion on tree branches [24].

#### 2.5.4. Interactive Adjustment Tools

After the development and testing of our reconstruction algorithm, we argue thatany automatic reconstruction methods generally require extensive parameter tuning because of the variability in the quality of the original scans and because of diverse range of complexity in crown architectures. In turn, the question of the trade-off between the time spent adjusting parameters and improvement in resulting model accuracy (and how much accuracy is required) can be raised. Also, because TLS data acquisition process remains complex, imperfect datasets are common and are thus likely to pose difficulties in implementing automated processes [19]. Therefore, we propose an alternative that incorporates a certain degree of human input. This semi-supervised reconstruction process refers to user-assisted, interactive model manipulation and guidance (both in-process as well as post-processing options). The PypeTree user interface is designed around the notion of a visual tree model, which is composed of two types of primitives: a series of articulation spheres, which are connected by truncated cones (or tubes, the radii of which are defined by their enclosing spheres). The manipulation of a sphere directly affects its connected tubes; creating, moving, resizing or deleting one will result in the model transformation that one would intuitively expect.

#### 2.5.5. Synthetic Model Creator

In order to overcome the challenges of obtaining validation information from the scanned trees, and to overcome the challenges of obtaining validation information from the scanned trees, and to allow for the rapid evaluation of our reconstruction algorithm, we have devised a method to create artificial trees, which is fully integrated into the PypeTree environment. To be useful in our context, such models should possess the following two properties:

- they are structured as a simplified, but reasonably realistic tree perennial structure (*i.e.*, in allometric terms) for which we can collect structural measures for comparison and validation, and
- they use a point-sampling method that produces results approximating those of a real TLS (*i.e.*, surface sampling and small random perturbations, possibly due to technical flaws).

The first goal is met by using an L-System grammar [25], viz., a set of recursive rules where the symbols govern the evolution (or “growing”) of geometric elements. When the rules are iteratively applied, they allow the creation of complex tridimensional structures such as trees or other plants [26,27]. The basic geometric elements of our artificial model are truncated cones, which correspond to branch segments, with length and radius variation being governed by additional parameters. For instance, the single-rule grammar in Equation (1) would yield a simple Y-shaped structure after one iteration:

(1) F→F[←F][→F]

The F symbol governs the creation of a segment, while the ← and → symbols impose directional changes. The [and] symbols correspond to a stack mechanism to spawn new branches recursively. From these models (*i.e.*, from user defined parameters and simulation results) we can collect structural information for validation purposes. To provide stochasticity within the virtual structure, branching angles and segment sizes vary according to a normal distribution for which the means and variances are user-defined.

The second property is obtained with a surface sampling mechanism that is applied to every segment. A point is first uniformly sampled from the truncated cone-side surface, with the two caps excluded. To simulate measurement errors that depend upon the device or its operational environment, a random perturbation displaces it away from the surface, following an exponential law (*i.e.*, with an exponentially shrinking likelihood as the displacement grows). The density of points per unit of surface and the scale of the exponential perturbation are user-defined parameters. The set of points that results from this process can be interpreted as a sampling of the exterior surface of the tridimensional synthetic model with no occlusion. Occlusion can be artificially added by deleting groups of points manually. The point cloud can be stored as a set of 3D coordinates, ready to be used as an artificial target for PypeTree algorithms.

## 3. Reconstruction of a Synthetic Tree: First Level of Validation

In this section, we will detail the reconstruction process of PypeTree for a synthetic tree model with the following parameters (all of the size values are in metres):

- Rule: A → [↑ FA] + + + [→ FA], axiom: FA
- Angle mean: 30°; standard deviation (SD): 5°
- Initial segment length: 1
- Segment length scaling: 0.75; SD: 0.1
- Initial segment radius: 0.1
- Segment radius scaling mean: 0.75; SD: 0.1
- Number of iterations: 7
- Sampling density per surface unit: 2000
- Sampling surface deviation (exp. scale): 0.001

**Figure 1.**
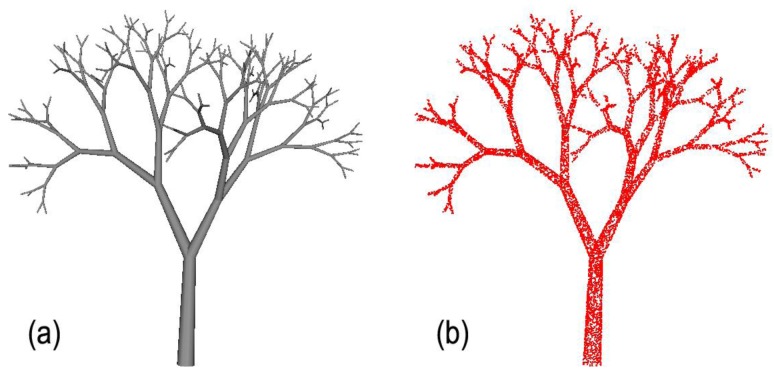
(**a**) 3D representation of the synthetic tree model that was created from L-System with 7 iterations. The model produced 256 segments and 128 tips; (**b**) 3D point cloud created from the synthetic model (13,474 points).

Given the aforementioned parameters, PypeTree generates the model that is illustrated in Figure 1a. The synthetic model occupies a 2.83 × 4.02 × 4.33 m volume and is the approximate size of a tree sapling. The model produced 128 tips and 256 segments, with the smallest being 0.13 m long. From this synthetic model, we produced a virtual point cloud of 13,474 points (Figure 1b) being representative of TLS scene produced from three scans after registration and raw data cleaning and filtering [8]. Average distance between points was 0.011 m in the point cloud that was created.

This synthetic model was designed to present a realistic and challenging model in terms of its size and branching structure. The sampling density for the point cloud that was created is lower than what raw data that are produced by a TLS instrument would yield, but this aspect actually makes the problem algorithmically harder because of the reduction of the number of points over objects minimal separation distance ratio.

### 3.1. Neighbourhood and Connectivity Repair Tool

**Figure 2.**
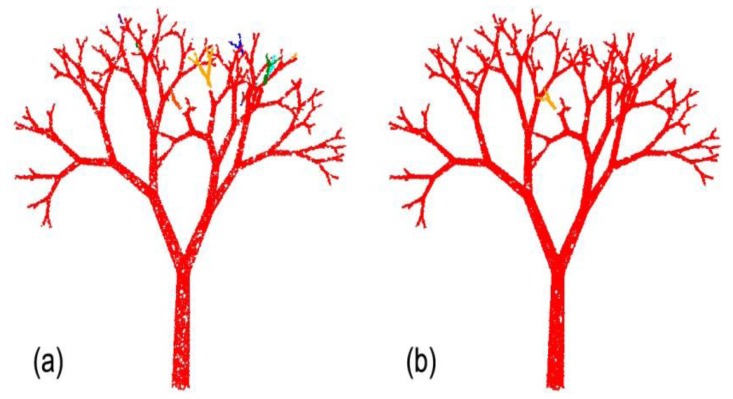

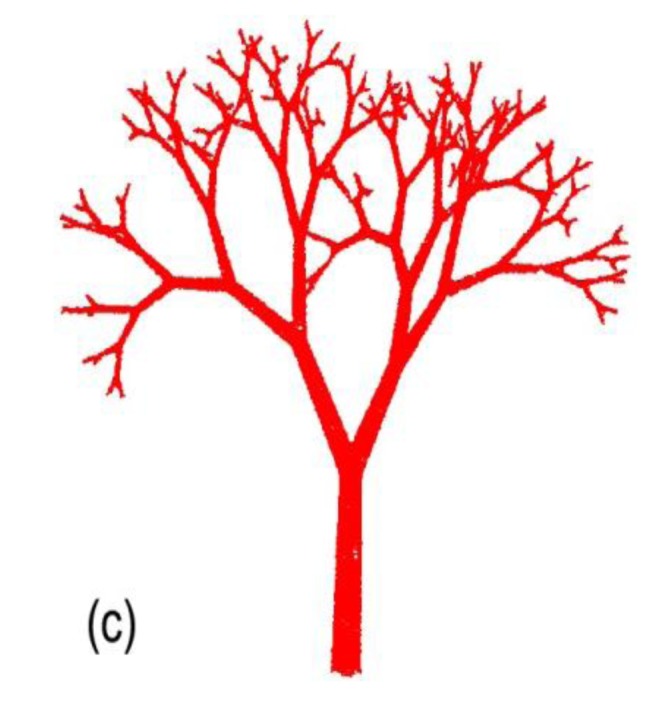
Effects of neighbourhood search radius *r* on *N*. Different colours refer to unconnected components (**a**) *r* = 0.04 (14 components); (**b**) *r* = 0.05 (two components) and (**c**) *r* = 0.06 (one component).

In Figure 2, we demonstrate how decreasing the value of *r* increases the fragmentation of *N* in different unconnected components and suggests *r* = 0.06 is the best, since it yields a single connected component for the whole tree.

**Figure 3.**
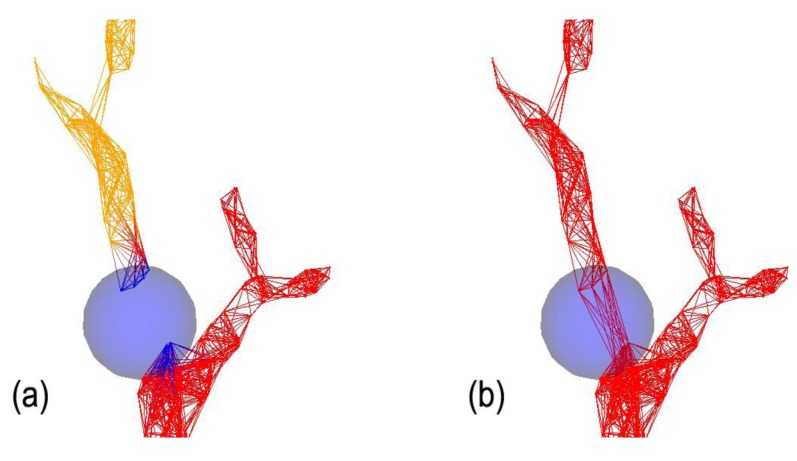
Close-up of a tree section (**a**) before and (**b**) after the assembly of two disconnected components using the Connectivity Repair Tool.

However, at this point the operator should validate that using this *r* value, certain fine features in the canopy are not fused together. If yes, a lower *r* value represents a better compromise that maintains branching sensitivity and quality, while creating several unconnected components (see Figure 2b). From here, the operator can use the connectivity repair tool by manipulating a “selection sphere” (Figure 3). This connection sphere can be used to force complete (many-to-many) connection of its enclosed points and provide a fully connected model, at an ideal density level.

### 3.2. Level Sets

**Figure 4.**
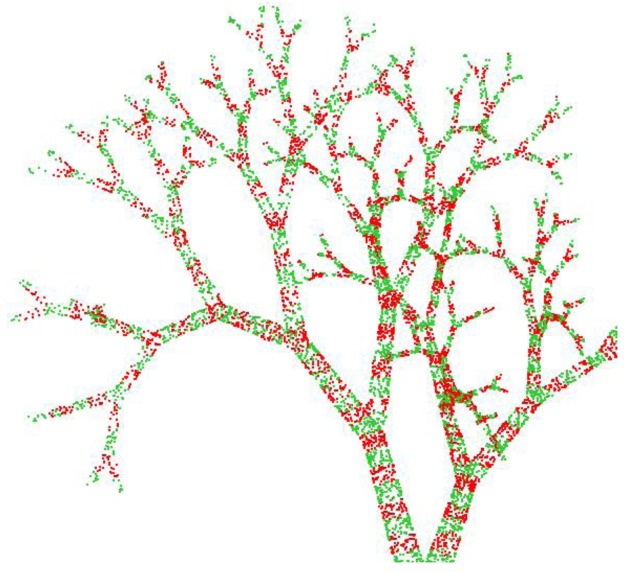
Point cloud segmentation using a level set value (*d*) of 0.09 m. Here, 50 levels were created and all tips (*i.e.*, 128) were detected.

The level sets are easy to compute on our synthetic model since the maximal value for *d* could be estimated as the smallest branch segment that is expected. For our virtual example, *d* is theoretically optimal at 0.13 m, and any value under 0.13 m will avoid ramification omissions and fit perfectly to the model (Figure 4). However, one should determine target minimum segment length and set *d* close to this value to prevent “false branch” creation.

### 3.3. Branch Manipulation Tools and Skeleton Smoothing

**Figure 5.**
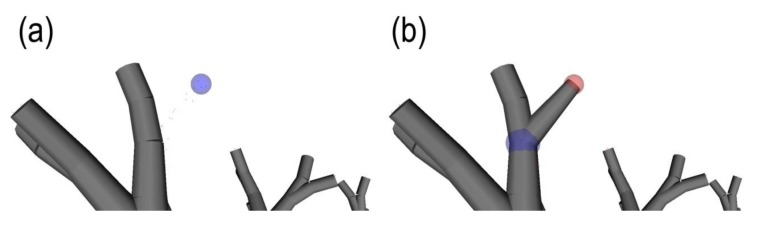
Close-up of a tree section (**a**) before and (**b**) after using the Branch Creation Tool.

In a close-up of the upper parts of the tree, Figure 5 illustrates the use of the branch creation tool. In this part of the crown, where disconnection that results from too large a value of *d* may occur, this tool allows the addition of tips or the connection of segments that have not automatically detected.

**Figure 6.**
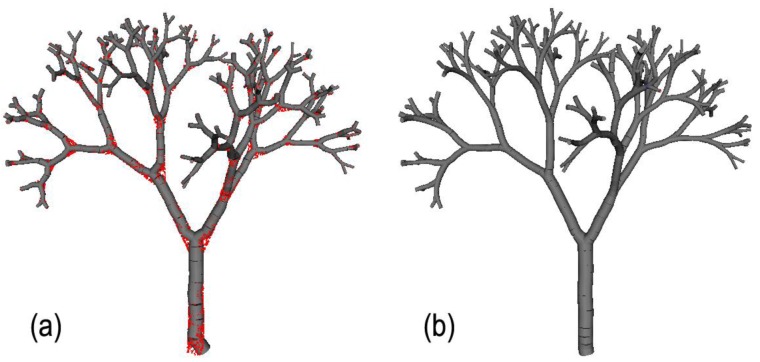
(**a**) Non-optimal reconstruction of the synthetic model (126 tips detected among the 128); (**b**) Perfect semi-supervised reconstruction (128 tips), using smoothing (*w* = 3) and the use branch creation tools.

Finally, Figure 6 summarises the gain in reconstruction quality (number of tips, branch insertion angles, and segment radii) that is made using the semi-supervised tools of PypeTree. First, we performed an initial reconstruction with a *d* value higher than the minimum segment length that produced 126 tips (Figure 6a). Second, we performed manual adjustments (i) for omitted segments using the branch creation tool; and (ii) shaped the tree skeleton using the smoothing tool, which can correct some branching point displacements, depending upon the level set settings that were chosen in previous steps (Figure 6b). Here, we consider the semi-supervised reconstruction as perfect by using the proportion of retrieved branch tips. This proxy is acceptable for complex branching structure, since perfect detection of the number of tips refers to perfect detection of ramification, which includes segment length and branching rate.

## 4. Reconstruction of Real Trees: Second Level of Validation

**Figure 7.**
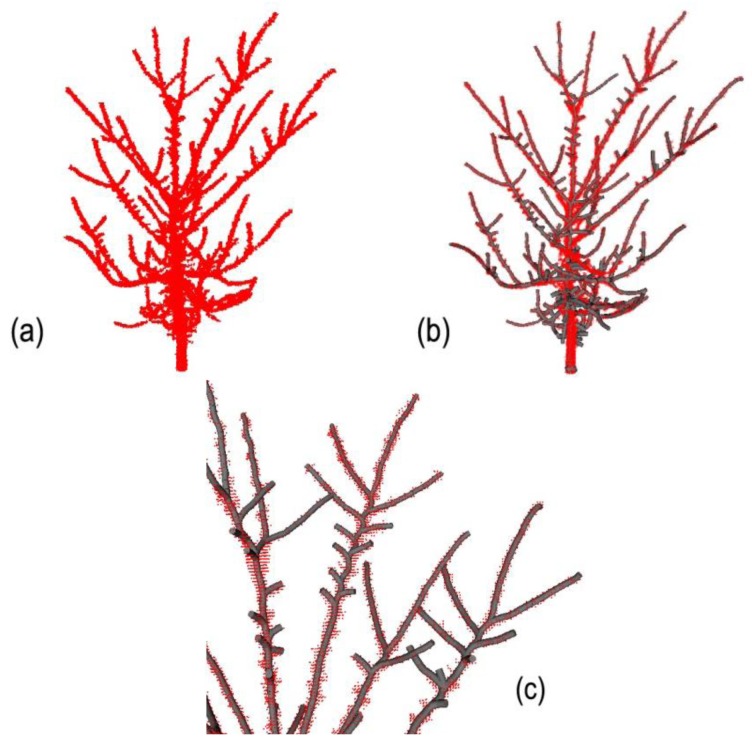
Automatic elm reconstruction. (**a**) Original point cloud from TLS (120,000 points); (**b**) Automated reconstruction using the following parameter combination: *r* = 0.025; *d* = 0.025; *w* = 3; (**c**) Close-up of the elm canopy showing the detection of the long axis but omission of some short second-order axes.

In this section, we first report the reconstruction of an individual elm (*Ulmus americana* L.) of 2.5 m height, from a point cloud (acquired with a TLS device Ilris-3D, Optech, Vaughan, ON, Canada) of 120,000 points with a minimal 0.01 m distance between points (Figure 7a). To obtain this scene and minimise occlusion, four scans were performed from four contrasting directions during leaf-off conditions. This greatly helped in obtaining a more robust point cloud, but discontinuities still occurred in some inner parts of the tree after registration. To validate reconstruction, we counted all tips for long- and short-shoots of first-, second- and third-order axes (Table 1), following the classification method of Barthelemy and Caraglio [28]. To get an idea of the total cumulated length, we then calculated the mean length of each type of axis from a random subsampling of WHAT (Table 1). Figure 7 shows the automatic reconstruction of the tree, which allowed the detection of up to 90% of the 1st - and 2nd- order axis numbers, but only 25% of the smallest axis order (cf. Table 1, and Figure 7b,c). We have estimated, based on the mean length of each axis order, the automatic reconstruction that led to the reconstruction of 85% of total cumulated length. It worths noting that this value is certainly underestimated, since omitted objects are generally the smallest of their order, but we attributed to them the mean size of order type. Despite accuracy of axis detection comparable to that of other methods [29], our approach led to higher total length detection since only the smallest branches were omitted.

The use of supervised tools improved model reconstruction, mainly by (i) connecting components that were isolated because of occlusion (*i.e.*, gaps of 3 cm); and (ii) creating short branches. After three hours of work, reconstruction increased to 78% of total number of axes detected and 93% of total cumulated length (Table 1). Better reconstruction seems impossible due to point cloud quality, especially in the lower part of the tree where branches are severely tangled. The accuracy of semi-supervised reconstruction makes it suitable for botanical or architectural studies, such as the investigation of the exploration (long axes) *vs*. exploitation (short axes) trade-off in trees [28]. One should acknowledge, however, the expense of time that was associated with this gain. When the aim of extracting tree metrics such as tree height and diameter or crown volume are for forestry purposes [8,17], this precision is obviously not necessary and the automatic method remains appropriate.

**Figure 8.**
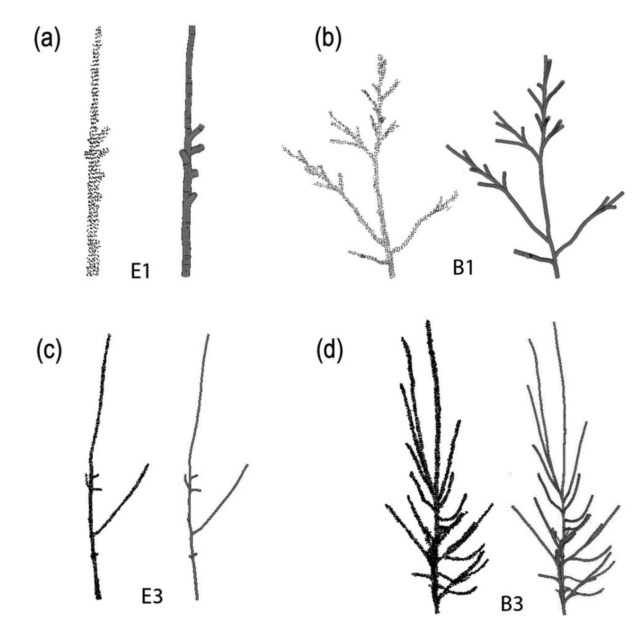
Reconstruction of 3-year-old saplings grown under contrasting light regimes. (**a**) E1: sugar maple under low light reconstructed using *k* = 10, *r* = 0.03, *d* = 100 and *w* = 5; (**b**) E3: sugar maple under high light reconstructed using *k* = 4, *r* = 0.03, *d* = 90 and *w* = 5; (**c**) B1: yellow birch under low light reconstructed using *k* = 5, *r* = 0.02, *d* = 30 and *w* = 3; (**d**) B3: yellow birch under high light reconstructed using *k* = 7, *r* = 0.01, *d* = 100 and *w* = 5.

In a second validation exercise, we have tested PypeTree on the reconstruction of a series of sugar maple (*Acer saccharum* Marsh.) and yellow birch (*Betula alleghaniensis* Britt.) individuals that were grown in a nursery under different light levels for three years (Figure 8). Scan settings and registration procedures were the same as the aforementioned elm tree. It should be noted that sugar maple architecture is less ramified than that of yellow birch, and a reduction in the amount of light that is available during growth tends to decrease ramification further. For this exercise, we have taken care to identify each segment, its position with respect to the parent axes, and its length, to allow precise quantitative validation. Two main validation parameters were then used to evaluate the quality of the reconstruction: (i) axis detection; and (ii) cumulative length detection.

Validation results for the automatic reconstruction are presented in Table 2 and show that axis omission varies between 15.8% and 42.9%. These values are rather high with respect to axis detection. However, these missing axes accounted for little in total individual length since the error on cumulative length detection only ranged between 2.2% and 9.1% (Table 2). Overall, mean detection error reached 25.6% and 5.1%, for axis and length detection, respectively. Moreover, reconstruction quality reached very high standards using the supervised adjustment tools. With less than 1 h of work per tree, several small branches were successfully added, decreasing axis omission by more than half (*i.e.*, 12.3%). In the case of E1, perfect axis detection was achieved with semi-supervised reconstruction (Table 3, Figure 8). The error on cumulative length detection was consequently kept under 3.2%, with a mean of 1.8% for the four trees (Table 3).

This validation exercise confirms that the length of certain branches was under the threshold imposed by the level sets parameter (*d*), but could be detected by the operator. According to these results, the overall complexity of a tree structure does not seem to affect automatic branch reconstruction. Segment length is clearly the limiting factor and 90% of the omitted axes in our validation exercise were under 3.5 cm length (data not shown). Considering the relatively simple architecture of these trees, if semi-supervised reconstruction did not allow for the detection of these small branches, only an increase in point cloud quality (*i.e.*, via TLS device improvement) may allow their detection.

In summary, whether the method was semi-supervised or not, PypeTree allows very high quality reconstruction. It does miss some small axes with lengths generally under 3.5 cm, but the importance of these axes is minimal in the total cumulated skeleton length of a tree. PypeTree is thus currently very adequate for an accurate reconstruction of seedlings and saplings. Recently, PypeTree was used successfully in the reconstruction and extraction of traits for isolated branches in an ice accretion study [24]. PypeTree is potentially adequate for bigger trees and more complex architectures, but only with the help of higher quality scans (high resolution TLS devices) or modelling for the finest parts.

## 5. Software Notes

PypeTree is written in Python, using the VTK [30] visualisation toolkit, as well as the NetworkX [31] and SciPy [32] libraries (for graph algorithms and *k*-*d* tree-based nearest-neighbour searches). The reconstruction process that has been discussed in this article is integrated into the user interface as a “wizard,” *i.e.*, a set of stage-specific window dialogs, within which it is easy to navigate back and forth, to study and modify the effects of certain parameter configurations. This modular organisation of the programme was designed with the goal of easing the integration of other reconstruction methods and tools that can be added or simply replace any step of the reconstruction. In addition, PypeTree is open-source software and possible collaborations and improvements by the user community are encouraged.

## 6. Conclusions

We have studied an existing reconstruction technique and improved it in the context of tree perennial tissue modelling and analysis. In addition to some core algorithmic modifications to augment its accuracy, enhancements include some operator-assisted tools built in the visual interface of our PypeTree programme, as well as a “virtual tree builder” allowing testing efficiently and validating programme modifications. We have shown that the use of this platform can result in high quality reconstructions for small trees and isolated branches, but still required a good knowledge of the studied target (*i.e.*, mainly the minimum expected branch length) and, in some cases, a supervised effort. PypeTree is thus well suited for the study of tree perennial tissue volume and development, crown volume and organisation, as well as botanical issues that are based on axis ordering. When coupled with allometric relationships or modelling exercises to replace foliage, PypeTree can also explore issues concerning the 3D functioning of trees (e.g., light interception, water transport and transpiration).

We thus argue that based on present TLS device precision: (i) the development of a purely automatic method to reconstruct very high quality models remains a challenging problem (even on saplings) due to size limitations of some tree axes and (ii) the effort that is spent in developing algorithms and tuning the parameters of a purely automatic method may be balanced by the use of well-designed supervised adjustment tools. Of course, automatic methods are obviously suitable for specific uses such as extracting rough tree metrics for forestry purposes. However, building on our findings, our next objectives would be to study the effects of ever-improving TLS sensing technology on PypeTree reconstruction capabilities.

## Figures and Tables

**Table 1.** Validation results for the elm tree reconstruction. Real data were acquired in the field and compared to automatic reconstruction and semi-supervised reconstruction. Both the number of segments and the cumulated estimated length of all segments were used to validate the reconstructions. Axis ordering system is based on Barthelemy and Caraglio [28].

**Axis Order**	**Real Elm Treemeasurements**			**Automatic Reconstruction**		**Semi-Supervised Reconstruction**	

	**Number**	**Mean Length (m)**	**Cumulated Length (m)**	**Number**	**Cumulated Length (m)**	**Number**	**Cumulated Length (m)**
**1st**	29	0.59	17.34	28	16.74	29	17.34
**2nd**	74	0.23	17.33	67	15.69	72	16.86
**3rd**	132	0.04	5.56	85	3.58	110	4.63
**4th**	131	0.02	3.63	34	0.94	75	2.08
**Total** (**% of real tree**)	366 (100%)		43.87 (100%)	214 (58%)	36.96 (85%)	286 (78%)	40.92 (93%)

**Table 2.** Validation results for the automatic reconstruction of 3-year-old sugar maple (E1 and E3) and yellow birch (B1 and B3) saplings that were grown under contrasting light regimes. Axis ordering system is based on Barthelemy and Caraglio [28].

**Tree ID**	**Axis**				**Cumulative Length** ^	

	1st Order M/R *	2nd and 3rd Order M/R *	Total M/R *	**Error %**	Cm M/R *	**Error %**
**E1**	7/3	0/0	7/4	42.9	79.5/72.3	9.1
**E3**	16/12	1/1	17/13	25.0	275.5/263.7	4.3
**B1**	16/13	13/10	29/23	18.8	420.5/401.4	4.6
**B3**	19/16	19/15	38/31	15.8	1219.0/1193.3	2.2
**Mean error**				**25.6**		**5.1**

* M states are for information that was directly measured on the sapling, while R states are for information that was extracted from the PypeTree reconstructions; ^ for this validation exercise, the actual length of each axis was measured.

**Table 3.** Validation results for the semi-supervised reconstruction of 3-year-old sugar maple (E1 and E3) and yellow birch (B1 and B3) saplings that were grown under contrasting light regimes. Axis ordering system is based on Barthelemy and Caraglio [28].

**Tree ID**	**Axis**		**Cumulative Length** ^	

	Total M/R *	**Error %**	Total M/R *	**Error %**

**E1**	7/7	0.0	79.5/80.0	0.6
**E3**	17/14	21.4	275.5/266.7	3.2
**B1**	29/25	16.0	420.5/411.0	2.3
**B3**	38/34	11.8	1219.0/1206.7	1
**Mean error**		**12.3**		**1.8**

* M states were for information that was directly measured on the sapling, while R states were for information that was extracted from the PypeTree reconstructions; ^ for this validation exercise, the actual length of each axis was measured.

## Notes

**Authors Contributions:** Sylvain Delagrange provided the original idea for the use and modification of the V&L algorithm to reconstruct tree perennial tissues. Sylvain Delagrange took the leadership on the manuscript writing and was responsible for the design and the analyses of the validation procedure. Christian Jauvin worked on the design and research of the reconstruction algorithm. Christian Jauvin developed the PypeTree software, as well as its semi-supervised approach. Pascal Rochon had a significant contribution in the development of several reconstruction steps and was in charge of TLS data acquisition and extraction for validation purpose.
